# Lung cancer metastasis to the scapula and spine: a case report

**DOI:** 10.1186/1746-1340-16-8

**Published:** 2008-08-12

**Authors:** James Demetrious, Gregory J Demetrious

**Affiliations:** 1 Private practice, Wilmington, NC, USA; 2 Post-graduate faculty, New York Chiropractic College, Seneca Falls, NY, USA; 3 Private practice, Wilmington, NC, USA

## Abstract

**Background:**

The objective of this case report is to describe the clinical presentation of a patient who complained of shoulder pain and was diagnosed with carcinoma of the scapula and spine that metastasized from the lung.

**Case presentation:**

A 76-year-old man without a history of cancer sought chiropractic care for right shoulder pain. Careful evaluation, radiographs, and subsequent imaging revealed primary and metastatic lung cancer. The patient was referred to his primary care physician for immediate medical care. Diagnostic images are included in this case to provide a comprehensive depiction of the scope of the patient's disease.

**Conclusion:**

Musculoskeletal symptoms are commonly encountered in chiropractic practice. It is important to recognize that primary lung cancer may be unidentified, and musculoskeletal symptoms may reflect the first sign of primary or metastatic pulmonary disease. Thoughtful evaluative procedure and clinical decision making, combined with the use of appropriate diagnostic tests may allow timely identification of primary or metastatic disease.

## Background

In the USA, more people die from lung cancer than any other type of cancer [1]. This is true for both men and women. In 2004, lung cancer accounted for more deaths than breast cancer, prostate cancer, and colon cancer combined [2].

Lung cancer can metastasize to virtually any bone, although the axial skeleton and proximal long bones are most commonly involved [3]. The primary symptom resulting from bone involvement is pain, which may have a pleuritic component when the ribs are involved. Bone pain is present in up to 25% of all patients at presentation [3].

Patients commonly seek chiropractic care with musculoskeletal complaints [4,5]. Through history and examination, chiropractic physicians have an opportunity to assess patients and determine whether serious conditions are present that may necessitate medical referrals.

Patients with previously identified or yet to be identified cancer may seek care with chiropractic physicians. This case report demonstrates previously undiagnosed lung cancer with widespread metastatic foci.

## Case presentation

### Case report

A 76-year-old male sought chiropractic care for complaints of right shoulder pain and mild right arm weakness. The onset of pain was insidious and of one week's duration. Pain was rated 8/10 on a visual analogue scale (0 = no pain, 10 = the worst pain of one's life). The pain was described as severe and worsened with movement. Additional symptoms included mild shortness of breath and posterior thoracic pain on respiration.

The patient's past medical history included headache, degenerative joint disease affecting the cervical spine, and a benign thyroid nodule. The patient reportedly smoked tobacco products for 50 years. He was a retired electrician.

The patient was afebrile. Vital signs were normal. Respirations were 18 cycles per minute. The lungs were clear to auscultation. The patient reported upper thoracic pain on inspiration.

A non-tender, mild decrease in active range of motion of the cervical spine was noted in all planes. No tenderness was elicited on palpation of the cervical spine. Cervical compression and Soto-Hall tests were negative. Valsalva maneuver was negative. Neurologic examination revealed no focal deficits.

Examination of the right shoulder revealed exquisite tenderness on palpation of the lateral border of the scapula with muscle spasm affecting the ipsilateral infraspinatus, teres major, and teres minor muscles. Active ranges of shoulder motion were restricted and painful in abduction, internal, and external rotation.

**Figure 1:**
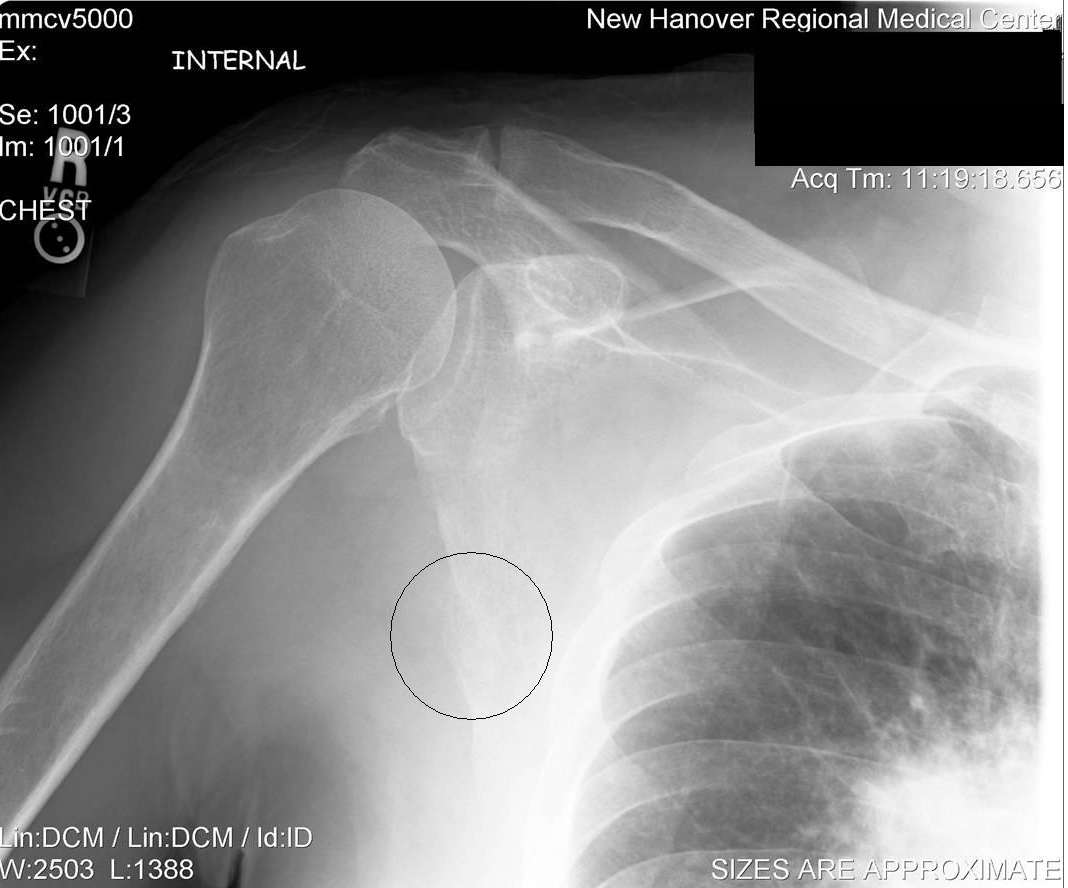
**AP radiograph of the right scapula reveals a focal indistinct lucency and lytic destruction of the lateral scapular cortical margin**.

Plain film radiographs of the right shoulder (AP with internal and external rotation views) and thoracic spine (AP and lateral views) were performed. Disruption of the cortical margin of the lateral border of the right scapula was noted as evidenced by an indistinct lucency (see Figure 1). In addition, a suspicious mass was noted in the hilar region of the right lung. Complete loss of the right hilar vascular detail secondary to the tumor mass effect were noted with visualized subsegmental infiltrate densities. No evidence of pleural effusion was noted.

The initial diagnostic impression included: suspicious right lung pathology and apparent lytic process affecting the scapula of an unknown origin. The patient was referred for imaging evaluations that included chest x-ray (CXR) and computed tomographic (CT) evaluation of the chest. He was referred to his primary care medical physician.

**Figure 2:**
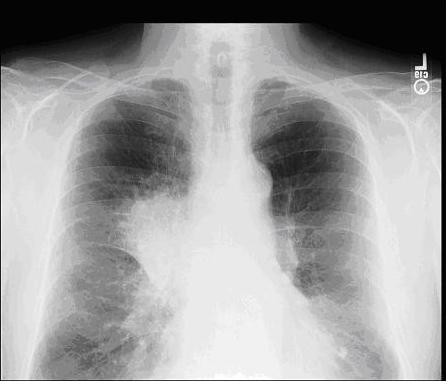
**PA chest radiograph reveals a right hilar mass**.

**Figure 3:**
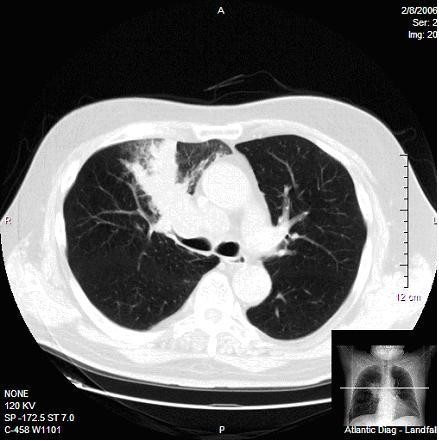
CT of the chest reveals a large mass in the right upper lobe of the lung with associated mediastinal and hilar adenopathy.

**Figure 4:**
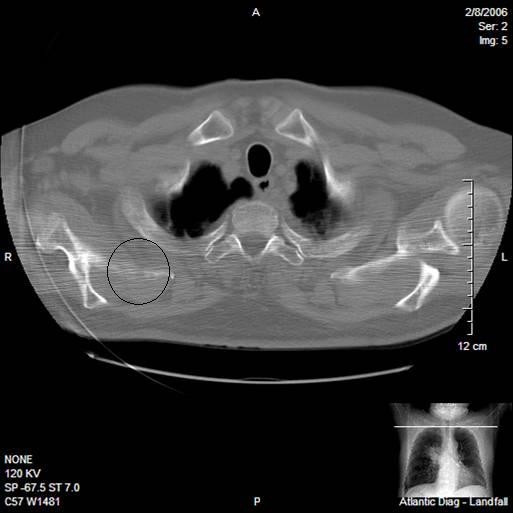
**CT of the chest reveals cortical lucency, expansile destruction, and medullary invasion due to metastatic lung carcinoma affecting the right scapula**.

The CXR and CT examination of the chest, abdomen and pelvis revealed:

1. A large mass in the right upper lobe of the lung with associated mediastinal and hilar adenopathy (see Figures 2 and 3).

2. Metastatic disease of the scapula (see Figure 4).

3. Metastatic liver disease.

**Figure 5:**
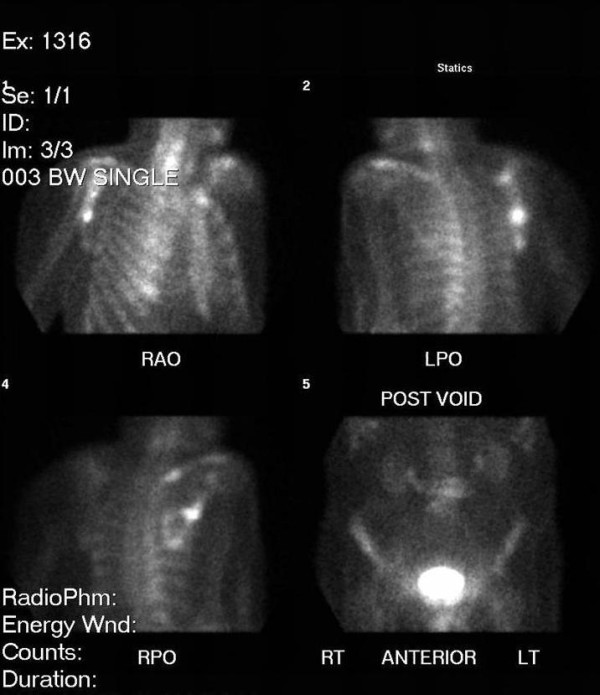
**Bone scintigraphy of the right scapula reveals increased uptake where metastatic lung carcinoma is present**.

**Figure 6:**
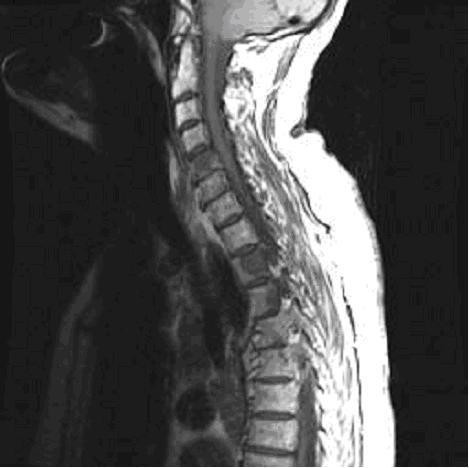
MRI sagittal T1WI reveals scattered foci of decreased signal intensity reflective of metastatic disease affecting the cervical and thoracic spine regions.

**Figure 7:**
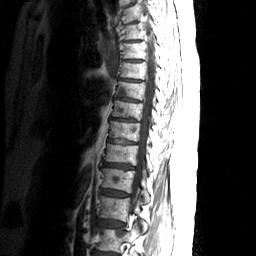
**MRI sagittal T1WI reveals scattered foci of decreased signal intensity reflective of metastatic disease affecting the thoraco-lumbar spine**.

Subsequent bone scintigraphy revealed abnormal increased accumulation of radiopharmaceutical along the lateral aspect of the right scapula (see Figure 5). MRI evaluation revealed additional metastatic foci including the cervical, thoracic and lumbar spinal regions as evidenced by multiple regions of decreased signal intensity are visualized on T1 weighted images (see Figures 6 and 7). Biopsy confirmed a primary lung carcinoma origin. Unfortunately, the patient succumbed to the disease within 3 months of its diagnosis.

## Discussion

### Chiropractic considerations

The identification of primary or secondary metastatic cancer requires careful consideration with regard to history and physical examination. A key objective for the chiropractic physician is to identify "red flags" as quickly as possible. This is especially true for any disease process that may weaken bone.

The application of directed force into spinal or osseous structures inherent to the chiropractic adjustment mandate careful evaluative procedure. Janse defined the adjustment as a specific form of articular manipulation using long or short lever techniques with specific contacts and is characterized by a dynamic thrust of controlled velocity, amplitude and direction [6].

While chiropractic physicians are challenged with the responsibility of attempting to identify relative and absolute contraindications to spinal adjustments, sometimes early onset, insidious and seemingly innocuous symptoms may delay early identification [7,8].

### Clinical considerations

When primary cancer is not yet identified, metastatic extension to skeletal structures can at times be difficult to detect [7,8]. As was illustrated in this case, clinical considerations that may assist or delay the identification of metastatic bone disease include:

1. Early in the course of the disease progression, important red flag identifiers may not initially be present and can delay early identification.

2. Initial pain presentations may be suggestive of common clinical conditions that are less aggressive.

3. Patients may or not be aware of, or report, the existence of a primary cancer.

4. Pain can be initially mild to severe and is often progressive in nature and unremitting despite therapeutic interventions.

5. It is sometimes extremely difficult to positively identify metastatic disease due to complex clinical factors [7,8].

Red flag indicators for metastatic bone disease include: age over 50 or under 20 years, a history of cancer, constitutional symptoms including unexplained weight loss, pain worse at night or in atypical areas, no significant improvement after > 1 month of conservative (non-invasive) care, pain that has no mechanical exacerbating or remitting factors, and severe disabling pain affecting a child or adolescent [9].

### Diagnostic imaging considerations

Humphrey reported that about 25% of people with lung cancer do not have symptoms from advanced cancer when their lung cancer is found [10]. Maghfoor reported that 7–10% of patients with lung cancer are asymptomatic and their cancers are diagnosed incidentally after a CXR was performed for other reasons [11]. Numerous studies have shown that the chest radiograph lacks sensitivity in detecting mediastinal lymph node metastases and in detecting chest wall and mediastinal invasion [12].

CT has become the major imaging modality of choice in the evaluation of patients with bronchogenic carcinoma [13]. Traditionally, chest CT for staging of lung cancer is extended into the abdomen to include the adrenal glands. Whether this requires intravenous contrast material is debatable [13]. Patz et al. [14] concluded that contrast-enhanced CT extended to include the liver rarely adds to routine nonenhanced CT through the adrenal glands and does not influence management decisions.

The evaluation of the mediastinum with magnetic resonance imaging (MRI) is approximately equal to that of CT with regard to the staging of bronchogenic carcinoma and MRI is significantly more accurate for detecting direct mediastinal invasion [15]. Other studies have confirmed the usefulness of MRI, particularly in the evaluation of chest wall invasion and the local staging of superior sulcus tumors [16,17]. The general conclusion of these studies is that MRI has advantages in the assessment of both chest wall and mediastinal invasion [13].

Indications for the use of whole body positron emission tomography imaging in lung cancer using 18-fluorodeoxyglucose (FDG-PET) in patients with non-small cell lung cancer include high clinical index of suspicion of high grade malignancy and radiographic evidence of nodal enlargement [13]. In addition, PET scans may be helpful in centers where mediastinoscopy is not readily available and in patients with significant comorbid conditions who are borderline candidates for surgery, with locally advanced disease, solitary brain metastasis, and cases of local recurrence that might qualify for reoperation [18,19].

Bone scintigraphy in the detection of metastatic disease has significant limitations. Although it has high sensitivity, it is noted for having very low specificity that ranges from 50%–60% [13]. Bone scintigraphy should probably be limited to cases in which patients have specified clinical indicators of bone metastasis [20].

When evaluating suspected pulmonary metastasis, CXR and CT of the chest are rated by the American College of Radiology (ACR) scale as: "9 – most appropriate" (Rating Scale: 1-Least appropriate, 9-Most appropriate) [21]. It is generally accepted that chest radiography, with posteroanterior (PA) and lateral views, should be the initial imaging test in patients without known or suspected thoracic metastatic disease [22-24]. Compared with chest radiography, CT is much more sensitive for detecting pulmonary nodules, because of its lack of superimposition and its high contrast resolution [22-24].

## Conclusion

Lung cancer is a significant and aggressive primary cancer with a predilection for skeletal metastasis. When primary lung cancer is not previously identified, metastatic disease to skeletal structures may initially manifest as musculoskeletal complaints. Careful diagnostic evaluation and decision making may allow for earlier diagnosis.

## Competing interests

The authors declare that they have no competing interests.

## Authors' contributions

JD conceived the study and drafted the manuscript. GJD participated in the care of the patient and provided data related to the case. Both authors read and approved the final manuscript.
